# Calcitonin Gene-Related Peptide (CGRP) Receptors Are Important to Maintain Cerebrovascular Reactivity in Chronic Hypertension

**DOI:** 10.1371/journal.pone.0123697

**Published:** 2015-04-10

**Authors:** Zhenghui Wang, Belén Cantó Martorell, Thomas Wälchli, Olga Vogel, Jan Fischer, Walter Born, Johannes Vogel

**Affiliations:** 1 Institute of Veterinary Physiology, University of Zürich, Zürich, Switzerland; 2 Group of CNS Angiogenesis and Neurovascular Link, and Physician-Scientist Program, Swiss Center for Regenerative Medicine, University of Zürich, and Divisions of Neurosurgery and Surgical Research, University Hospital of Zürich, Zürich, Switzerland; 3 Division of Neurosurgery and Laboratory of Molecular Neurooncology, University Hospital Zürich, Zürich, Switzerland; 4 Brain Research Institute, University of Zürich, and Department of Health Sciences and Technology, Swiss Federal Institute of Technology (ETH) Zürich, Zürich, Switzerland; 5 Research Laboratory for Calcium Metabolism, Orthopedic University Hospital Zürich, University of Zürich, Zürich, Switzerland; University of North Dakota, UNITED STATES

## Abstract

Cerebral blood flow autoregulation (CA) shifts to higher blood pressures in chronic hypertensive patients, which increases their risk for brain damage. Although cerebral vascular smooth muscle cells express the potent vasodilatatory peptides calcitonin gene-related peptide (CGRP) and adrenomedullin (AM) and their receptors (calcitonin receptor-like receptor (Calclr), receptor-modifying proteins (RAMP) 1 and 2), their contribution to CA during chronic hypertension is poorly understood. Here we report that chronic (10 weeks) hypertensive (one-kidney-one-clip-method) mice overexpressing the Calclr in smooth muscle cells (CLR-tg), which increases the natural sensitivity of the brain vasculature to CGRP and AM show significantly better blood pressure drop-induced cerebrovascular reactivity than wt controls. Compared to sham mice, this was paralleled by increased cerebral CGRP-binding sites (receptor autoradiography), significantly in CLR-tg but not wt mice. AM-binding sites remained unchanged. Whereas hypertension did not alter RAMP-1 expression (droplet digital (dd) PCR) in either mouse line, RAMP-2 expression dropped significantly in both mouse lines by about 65%. Moreover, in wt only Calclr expression was reduced by about 70% parallel to an increase of smooth muscle actin (Acta2) expression. Thus, chronic hypertension induces a stoichiometric shift between CGRP and AM receptors in favor of the CGRP receptor. However, the parallel reduction of Calclr expression observed in wt mice but not CLR-tg mice appears to be a key mechanism in chronic hypertension impairing cerebrovascular reactivity.

## Introduction

In healthy subjects cerebral blood flow autoregulation (CA) ensures a relatively constant cerebral blood flow (CBF) during variations in arterial blood pressure between 50–150 mmHg. Consequently, impairment of CA allows CBF to either drop or rise passively during fluctuations of arterial blood pressure, which can result, next to syncope and falls, in severe cerebral ischemia or brain edema [1]. The clinical relevance of disturbed CA becomes obvious considering the fact that the major health problem in Western countries, hypertension, is associated with an impairment of the dynamic CA [2]. In patients suffering from chronic hypertension the lower limit of CA shifts towards higher pressures thereby making these individuals highly vulnerable to brain ischemia in response to anti-hypertensive therapy or when subjected to acute hypotension of other reasons [3]. For obvious reasons, the upper limit of CA has not been determined in normo- or hypertensive humans. In normotensive baboons, however, it is located between 120 and 150 mm Hg and between 155 and 170 mm Hg in chronic hypertensive baboons [4]. Although the alterations of CA in response to chronic hypertension have been described many years ago [5,6,7] the mechanisms behind remain poorly understood.

We hypothesize that expression changes of the receptors for calcitonin gene-related peptide (CGRP) and/or adrenomedullin (AM) play a significant role in the patho-physiology of CA during chronic hypertension because both peptides are the most potent vasodilatatory peptides known so far [8,9] and previous data suggest their involvement in CA [10,11,12]. The receptors for these peptides are heterodimers of the calcitonin receptor-like receptor (Calclr) and either the receptor-activity modifying protein (RAMP)-1 or -2 forming at the cell surface a receptor for CGRP or AM, respectively [13]. Of note, the Calclr might be constitutively expressed whereas the ligand specificity as well as the sensitivity of the cells might be under regulation by exchanging the associated RAMP [14]. All receptor components for AM as well as CGRP are naturally expressed in vascular smooth muscle cells [15] and vasodilatation of rat pial arteries in response to a stepwise hypotension is mediated, at least in part, by CGRP, which is released from perivascular sensory fibers. Accordingly, vasodilatation in response to hypotension is attenuated by CGRP receptor desensitization and after application of capsaicin, which results in depletion of CGRP from perivascular neurons [16,17]. On the other hand, AM levels are about 50% higher in the cerebral than in the peripheral circulation because cerebral endothelial cells secrete large amounts of AM [11]. Acutely administered AM is able to increase CBF in a dose dependent manner as measured in rats in superficial cortical layers with Laser-Doppler flowmetry [18].

Our data obtained in the present study suggest that increased Calclr signaling preserves cerebrovascular reactivity during chronic hypertension. RAMP-2 but not RAMP-1 expression is highly suppressed suggesting that in chronic hypertension, first, the receptor stoichiometry for CGRP and AM is shifted by expression changes of the RAMPs and, second, that compensatory mechanisms to maintain cerebrovascular reactivity during chronic hypertension rest rather on CGRP than on AM signaling. Moreover, in chronic hypertensive wt mice Calclr expression is also suppressed, which could be a so far underrated factor contributing to the reduced cerebrovascular reactivity in hypertensive patients.

## Methods

### Animals

Mice transgenic for the calcitonin receptor-like receptor (CLR-tg) overexpress a V5 (GKPIPNPLLGDST) tagged rat CLR under control of the mouse smooth-muscle α-actin promoter (Acta2) [19,20]. Non-transgenic littermates (BL6xDBA2) of CLR-tg mice served as wild type controls. The experiments in this study conformed to the 'European Convention for the Protection of Vertebrate Animals used for Experimental and other Scientific Purposes' (Council of Europe No 123, Strasbourg 1985) as well as institutional and local governmental guidelines and were approved by the Cantonal Veterinary Department, Zurich, Switzerland (Permit Number: 57/2008).

### Surgery

Chronic hypertension was induced in female mice using the one-kidney-one-clip (1K1C) model [21]. Briefly, mice aging 7 months were anesthetized with 4% Isoflurane in O_2_. Then the right renal artery was clipped with an U-shaped stainless steel surgical clamp (2 x 1 x 0.8 mm outer dimensions with a gap width of 0.12 mm and gap length of 1.5 mm, Exidel SA R&D microtechniques). In sham-operated animals no clip was placed. One week later the left kidney was removed under 4% Isoflurane anesthesia in O_2_. Before wound closure always 50000 U Benzypenicillin-Na/K (Penicillin 10 Mega, Grünenthal) dissolved in 0.9% saline were dropped into the wound. Prior to discontinuation of the anesthesia and twice a day for three subsequent days the mice were treated with 5mg/kg Flunixin s.c.. Initially we tested the effect of this surgery on mean arterial blood pressure 6, 8 and 10 weeks after the second operation. A significant increase of blood pressure was established 8 weeks after surgery and stayed constant thereafter (not shown). All subsequent experiments were therefore performed 10 weeks after surgery.

For measuring cerebral autoregulation (CA) [22] anesthesia was induced with 4% Isoflurane in O_2_. Then the mice were equipped with catheters in both femoral arteries and one femoral vein for the measurement of blood pressure, heart rate (PlugSys, Hugo Sachs Electronics, and PowerLab, ADInstruments), blood gases and acid base status (AVL700, Radiometer Medical), infusion of anesthetics (see below) and exsanguination. Before closing the wounds a drop of 2% Lidocain (Streuli) was applied to the wound. Thereafter the mice were placed in a stereotactic frame, the scull was exposed and flushed with 2% Lidocain. Then a Laser speckle perfusion imager (moorFLPI, Moor Instruments) was adjusted to its maximal magnification and five regions of interest (ROI’s, 3 on the left and 2 on the right parietal cortex) were defined avoiding visible vessels. In accordance with others [23] we found that the transparency of the scull of mice is high enough to not reduce significantly the laser signal as long as it is kept wet with artificial cerebrospinal fluid or 0.9% saline warmed to 37°C. The images were acquired at 25 Hz and the traces of the ROI’s were sampled with a time constant of 0.5 sec. For measurement of CA the anesthesia was changed to intravenous Etomidate (Etomidat Lipuro) at an infusion rate of 14–26μg/min as described [22]. The CA measurements that were commenced 20 min after changing the anesthesia and measurement of the arterial acid-base-status (ABL700, Radiometer Medical). During surgery and the subsequent measurements of static CA animals were allowed to breath spontaneously pure oxygen.

### Measurement of cerebral autoregulation CA [22]

At the very beginning of the recordings a screwdriver handle was dropped onto the arterial line connected to the blood pressure transducer. This produced an artifact simultaneously in both the blood pressure and the laser perfusion trace that was later used to synchronize both traces for offline calculation of the CA limits. Then the mice were slowly exsanguined via the second arterial line while continuously recording the laser speckle perfusion and blood pressure signal. The exsanguination rate was adjusted manually by continuous inspection of the blood pressure trace to get a linear and constant decrease of the blood pressure (about 1–2mmHg/min, total exsanguination time: 35–40min).

For determination of the CA limits all laser speckle perfusion and blood pressure values before the marker artifact were discarded and then the data were averaged in ten second intervals using macros written in Excel (Microsoft). The laser speckle perfusion values obtained in this way were plotted as a function of the corresponding blood pressure values. Then the lower CA limit was determined by linear extrapolation by starting with the lowest blood pressure values and the upper limit in a similar way by starting with the highest blood pressure values as described elsewhere for the “plateau constraint” [24]. This was done separately for each of the five ROI’s. The final CA limits for each animal were defined as the mean of those determined for each ROI and these mean values were used for statistical analysis. The intra-individual variation of the ROI’s was very low (coefficient of variation: 1.3–2.8%).

**Fig 1:**
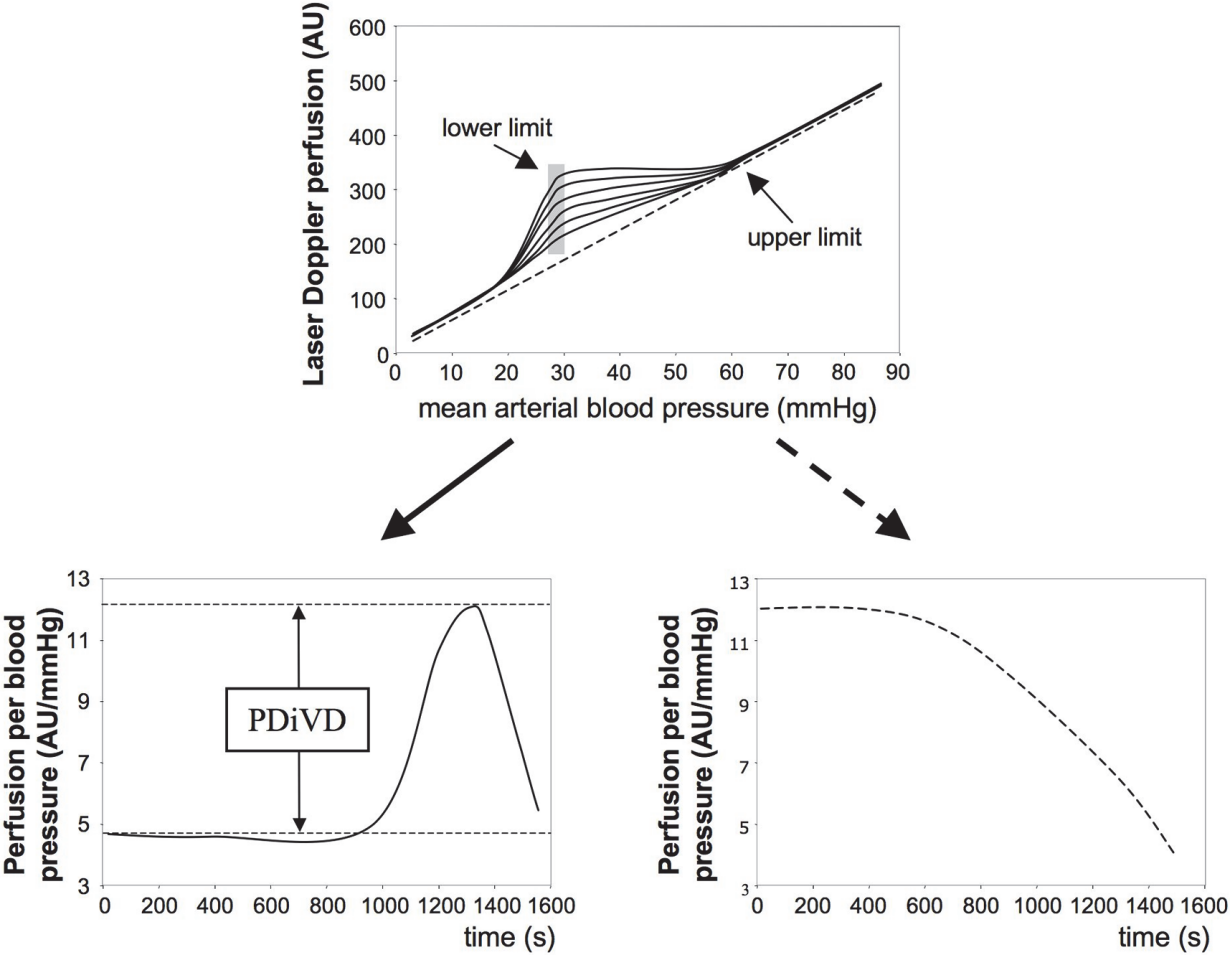
Definition of pressure drop-induced vasodilatation *(PDiVD)*. The upper panel shows normal CA curves (solid lines) with typical “knee”-shaped deviating from a linear correlation between blood pressure and perfusion that indicates completely lost CA (dashed line) [22]. The corresponding CA limits are indicated. The vertical gray shaded area demonstrates that the lower CA limit could stay at the same blood pressure value even when the CA curve would be flatter, meaning that the same lower CA limit would be associated with less perfusion. To quantify this, we plotted the time course of the perfusion per blood pressure of the CA measurement (lower panels). When CA is abrogated (dashed line in the upper panel) the perfusion/blood pressure passively decreases with time or falling systemic blood pressure (lower right panel), which is typical for elastic tubes. In contrast, with intact CA (solid lines in the upper panel) the perfusion/blood pressure starts to rise from a certain time point on, reaches a maximum and then falls again (lower left panel). We defined the difference between the initial perfusion/blood pressure value and the maximal one as PDiVD (horizontal dashed lines in the lower left panel).

In addition to determination of the classical CA limits we intended to better characterize the cerebrovascular reactivity by introducing a new way of analyzing the perfusion data obtained. To this end, for each time point from the moment on of starting the exsanguination the perfusion per mmHg was calculated, which we define as pressure drop-induced vasodilatation (PDiVD). This parameter is explained in Fig 1 and its legend.

After completion of the CA measurements the brain was removed and cut sagittally into two halves of which one was used for receptor autoradiography and the other for gene expression analysis.

### Receptor autoradiography for αCGRP and AM

αCGRP and AM binding sites in the brain were determined by receptor autoradiography using either ^125^I labeled αCGRP or AM (PerkinElmer) as described [25]. Briefly, 20μm thick cryosections were incubated for 3h at 4°C in a blocking solution containing 50mM Tris-HCl and 3% BSA, pH 7,4. Then this buffer was exchanged with the same buffer but now containing the radioactively labeled peptides (60 nCi / slide) and incubated overnight at 4°C. After washing away the buffer with distilled water the slides were air died and exposed to an X-ray film for two weeks. Unspecific binding was assessed by adding 1μM unlabelled αCGRP or AM, respectively, to the pre-incubation blocking solution.

Then, images of the X-ray films were acquired using a densitometry camera (CoolSNAP cf, Roper Scientific (Photometrics)) together with an ultra stable precision illuminator (Model R95 Northern light, Imaging Research). The resulting optical densities were quantified using an image analyzing system (MCID Analysis 7.0) after calibration with the gray values of ^14^C standards co-exposed on the same X-ray film. Beforehand, these ^14^C standards had been calibrated to assign each optical density a tissue concentration of ^125^I (nC/g).

### Gene expression analysis

RNA extracts (RNeasy, Qiagen) obtained after on-membrane DNase I digestion (Qiagen) of brain hemispheres were assessed for their quality by measuring the 280/260nm ratio and using the Agilent bioanalyzer (Agilent Technologies). Only samples with an A260/A280 ratio > 1.95 were accepted and RNA integrity number (IN) determined. These samples had an IN of 7.9 ±0.6 and were transcribed to cDNA (iScript, Bio-Rad). Always exactly 250ng RNA were transcribed and the resulting product was then diluted 1:100. Of this solution 9μl were taken with a high-precision pipette (Rainin) for quantification of target gene expression (cf. Table 1) using droplet digital PCR (ddPCR, Bio-Rad) according to the manufactures instruction. To this end, end-point PCR with 41 cycles was performed after splitting each sample into about 20000 droplets. Next the droplet reader (QX100, Bio-Rad) used at least 10000 droplets to determine the percentage of positive droplets and calculation of copy number per μl.

**Table 1:** TaqMan Gene Expression Assays used for ddPCR.

*Gene name*	*Gene Symbol*	*Reference sequence*	*Assay location*	*Applied Biosystems reference*	*Label*
calcitonin receptor-like receptor	Calcrl	http://www.ncbi.nlm.nih.gov/nuccore/NM_018782.2	1140	Mm00516986_m1	VIC
Receptor activity modifying protein 1 (variants 1–3)	RAMP-1	http://www.ncbi.nlm.nih.gov/nuccore/NM_016894.3, http://www.ncbi.nlm.nih.gov/nuccore/NM_178401.3, http://www.ncbi.nlm.nih.gov/nuccore/NM_001168392.1	321	Mm00489796_m1	FAM
Receptor activity modifying protein 2	RAMP-2	http://www.ncbi.nlm.nih.gov/nuccore/NM_019444.2	332	Mm00490256_g1	FAM
actin, alpha 2, smooth muscle	Acta2	http://www.ncbi.nlm.nih.gov/nuccore/NM_007392.3	1403	Mm00725412_s1	FAM
actin, beta	Actb	http://www.ncbi.nlm.nih.gov/nuccore/NM_007393.3	1230	Mm00607939_s1	VIC

As negative control transcription reactions of isolated RNA were also performed without reverse transcriptase. In addition, each primer / probe pair was tested on genomic mouse DNA and under these conditions none of them showed positive droplets. All expression analyses were performed in duplicate using mouse “best coverage” TaqMan kits from Applied Biosystems (cf. Table 1). The reproducibility of the measurement of copy numbers per μl was quite good (average coefficient of variation 12 ±8.8%).

### Statistics

Data were analyzed with the GraphPad PRISM 4 Software (version 4.01) using ANOVA and Students t-test or Kruskal-Wallis test (in case the number of values were not equal for all groups) for unpaired samples with Bonferroni’s or Dunn’s post hoc test respectively. P values of <0.05 were considered significant.

## Results

Table 2 shows the physiological variables measured just before commencing the determination of cerebrovascular reactivity. There were no differences between wt and CLR-tg. Mean arterial blood pressure and heart rate in sham animals were also not different between the genotypes and comparable to the values reported previously in untreated wt or CLR-tg mice [20].

**Table 2:** Physiological variables.

	*pH*	*pCO*_*2*_ *(mmHg)*	*pO*_*2*_ *(mmHg)*	*BE (mmol/L)*	*Hct (%)*
wt (n = 19)	7.32 ±0.07	35.8 ±4.7	433.7 ±110	-11.9 ±2.1	43.4 ±4
CLR-tg (n = 17)	7.32 ±0.11	34.1 ±12.5	424.4 ±88	-11.1 ±1.7	43 ±3.7

Values are means ±SD, wt = control mice, CLR-tg = calcitonin receptor-like receptor transgenic mice (BE = base excess, Hct = hematocrit).

**Fig 2:**
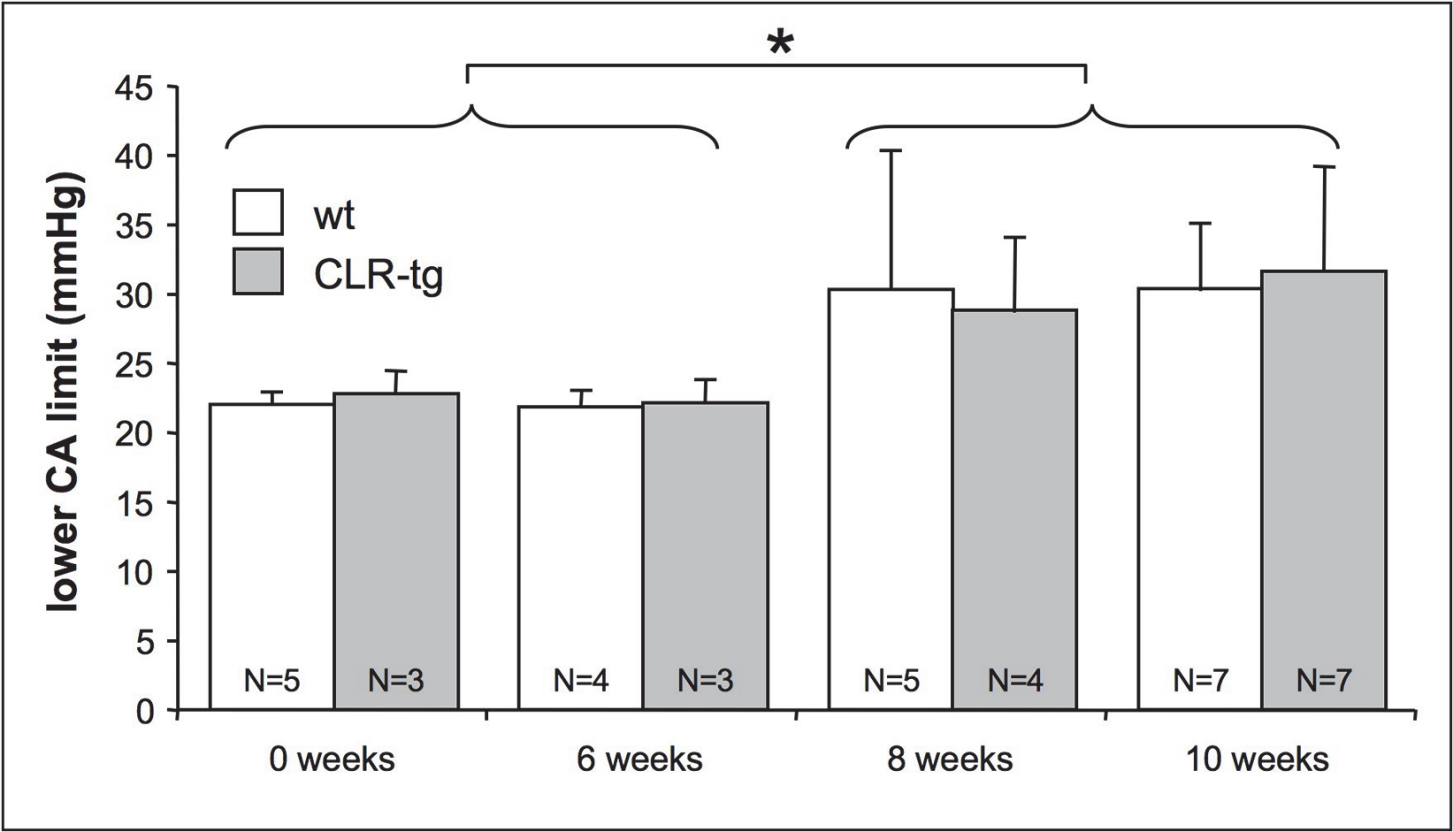
Time course of the upwards sift of the lower CA limit after 1K1C surgery. The lower CA limit shifted in both mouse lines in a similar fashion. Significantly elevated lower CA limits were not observed before 8 weeks of hypertension. Thereafter no further shift of the lower CA limit occurred. * = p<0.05 after pooling the data from 0 and 6 weeks and 8 and 10 weeks respectively. Means ±SD of as many animals as indicated.

The effect of the 1K1C surgery on mean arterial blood pressure was tested 6, 8 and 10 weeks later. A significant increase of the mean arterial blood pressure of about 30 mmHg in both lines was established 8 weeks after surgery and stayed constant thereafter. These mice were also subjected to measurement of the lower CA limit using the “plateau constraint” approach [24]. Similar to mean arterial blood pressure the lower CA limit was significantly increased by about 8–10 mmHg after 8 weeks of hypertension without further increase until the 10^th^ week (Fig 2). All subsequent experiments were therefore performed 10 weeks after surgery.

**Fig 3:**
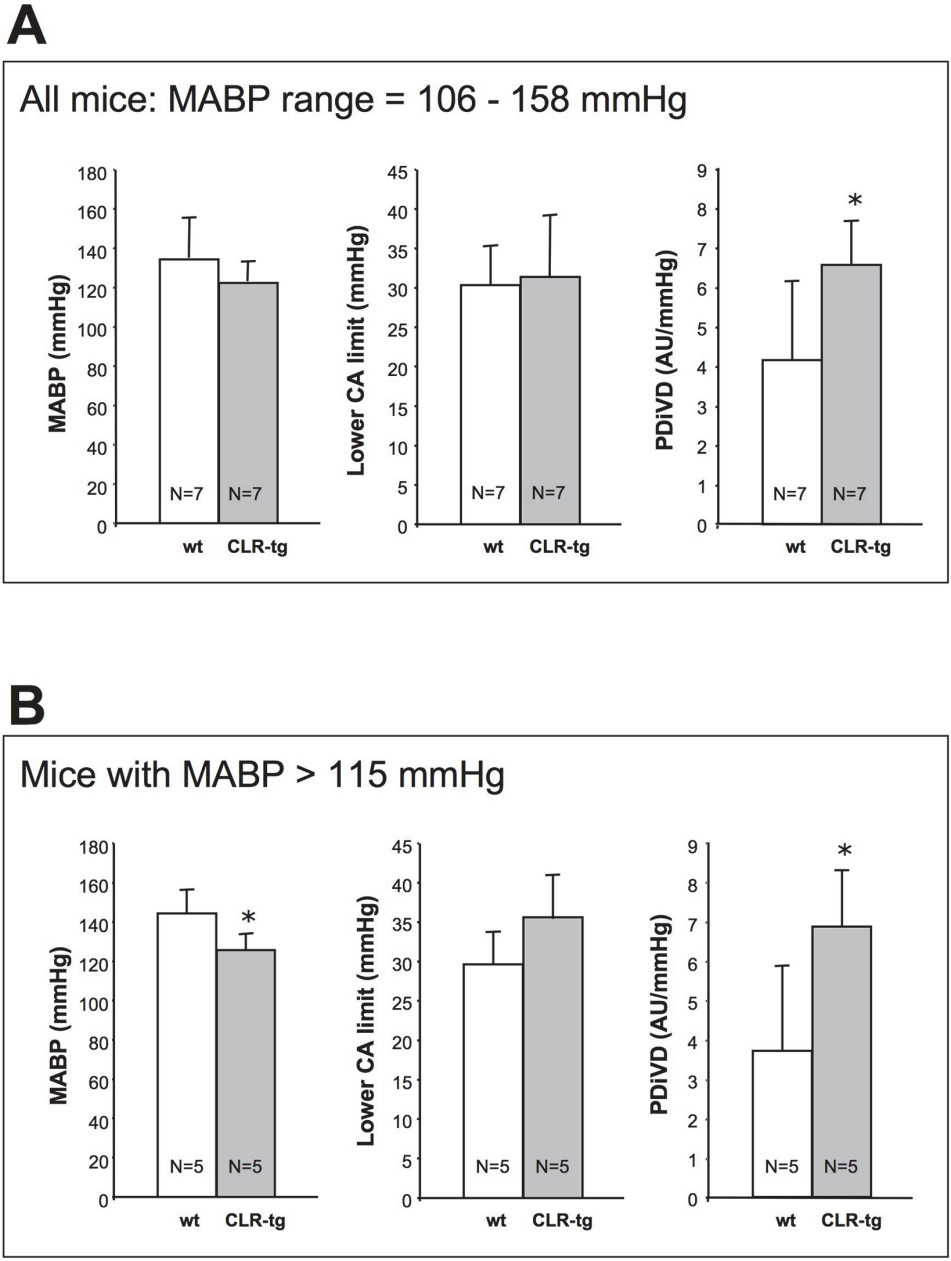
Assessment of cerebrovascular reactivity. Ten weeks after 1K1C surgery the systemic mean arterial blood pressure (MABP) of all animals ranged from 106 to 158 mmHg and did not differ between wt and CLR-tg mice (A, left bar graph). The lower CA limit was comparably shifted in both mouse lines (A, middle bar graph; cf. also Fig 2). However the PDiVD was significantly higher in CLR-tg compared to wt mice (A, right bar graph). When excluding two wt and two CLR-tg mice with a MABP below 115 mmHg, the CLR-tg showed slightly but significantly lower MABP (B, left bar graph), again no difference in the lower CA limit (B, middle bar graph) and an even more pronounced higher PDiVD (B, right bar graph). Means ±SD of as many animals as indicated.

Fig 3 shows the cerebrovascular reactivity after 10 weeks of hypertension. The final elevation in systemic mean arterial blood pressure (MABP) varied between 106 and 158 mmHg and was the same in wt and CLR-tg mice (Fig 2A, left bar graph). However when omitting animals with a MABP below 115 mmHg, which can be considered as pre-hypertensive according to the classification of the American Heart Association (AHA) given for humans [http://www.heart.org/HEARTORG/Conditions/HighBloodPressure/AboutHighBloodPressure/Understanding-Blood-Pressure-Readings_UCM_301764_Article.jsp] the CLR-tg mice showed a slightly but significantly lower MABP (Fig 2A, left bar graph) suggesting that hypertension in wt mice was marginally more severe. When comparing the shift in the lower limit of CA between wt and CLR-tg mice there was no difference independent of taking all animals into account or only those with a MABP above 115mmHg. In contrast, CLR-tg showed consistently higher PDiVD suggesting better cerebrovascular reactivity. Moreover these data also suggest that PDiVD appears to be a better parameter to detect differences in cerebrovascular reactivity compared to a shift of the lower CA limit.

**Fig 4:**
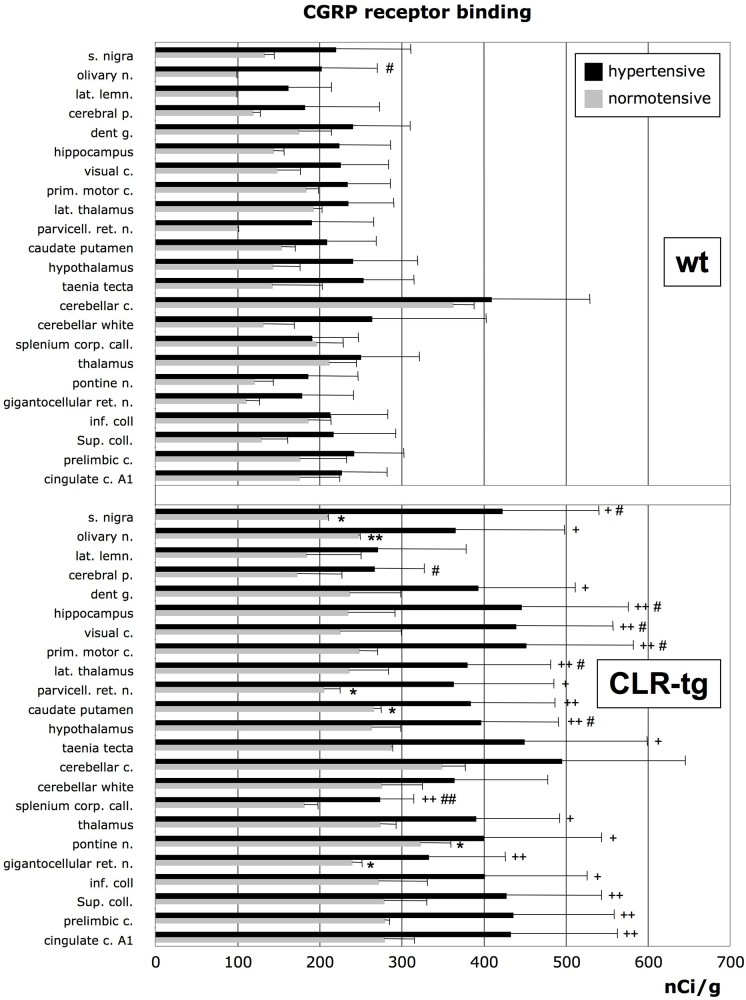
CGRP binding sites in different brain structures. Generally CGRP binding was much higher in CLR-tg mice. Moreover, in wt mice hypertension did increase CGRP binding non-significantly only by trend whereas in CLR-tg mice hypertension-induced increase in CGRP binding was more pronounced and significant (* = p<0.05 and ** = p<0.01 vs. normotensive wt mice, # = p<0.05 and ## = p<0.01 vs. normotension, + = p<0.05 and ++ = p<0.01 vs. hypertensive wt mice). Means ±SD of 7 animals per group.

Receptor autoradiography showed no significant effect of either genotype or hypertension regarding AM binding (mean of all investigated brain structures: wt_normorensive_: 160.2 ±48.3 nCi/g, CLR-tg_normotensive_: 178.1 ±27.5 nCi/g, wt_hypertensive_: 148.4 ±27.3 nCi/g, CLR-tg_hypertensive_: 161.6 ±33.3 nCi/g). In contrast, brain sections from CLR-tg mice showed considerably more CGRP binding (Fig 4). Compared to wt mice, after sham-operation as well as after 1K1C surgery CLR-tg mice showed 59% and 73% higher CGRP binding, respectively. 1K1C surgery-induced hypertension increased CGRP binding in wt as well as in CLR-tg mice (mean of all investigated brain structures shown in Fig 4: wt_normorensive_: 158.2 ±55.7 nCi/g, CLR-tg_normotensive_: 251.1 ±43.3 nCi/g, wt_hypertensive_: 225.9 ±48.1 nCi/g, CLR-tg_hypertensive_: 390.6 ±59.9 nCi/g). However, in wt mice this was significant just in 1 out of 23 brain structures suggesting a slight up-regulation of functional CGRP-receptors. In contrast, in chronic hypertensive CLR-tg mice 8 brain structures showed significant more CGRP binding compared to normotensive CLR-tg mice.

**Fig 5:**
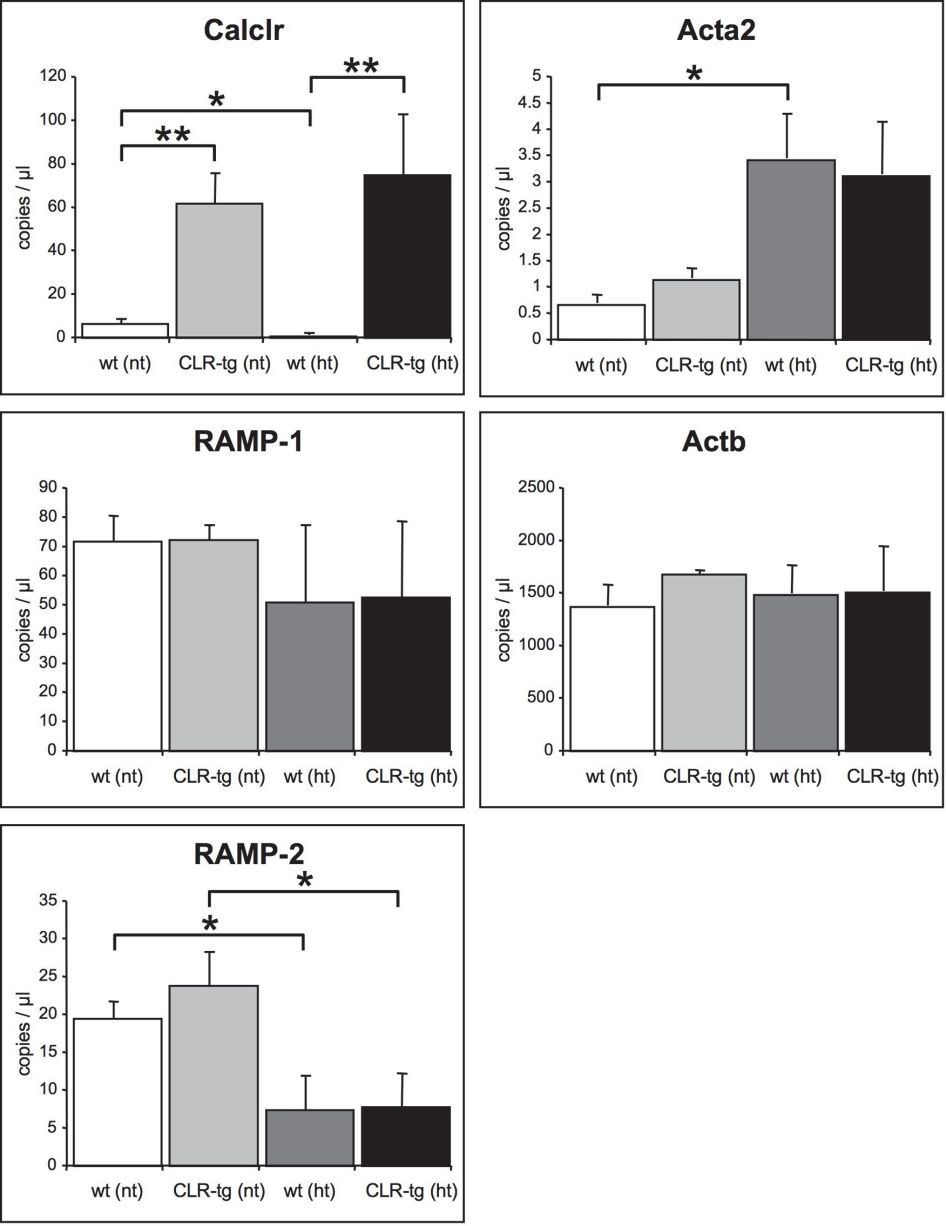
Gene expression analysis of brain tissue. The abundance of Calclr mRNA was due to the transgenic overexpression much higher in the CLR-tg mice (upper left panel). Hypertension induced a significant reduction of the Calclr expression by about 70% in wt mice whereas it slightly but non-significantly increased in CLR-tg mice most likely due to the increased expression of Acta2 (upper right panel) as the transgene of the CLR-tg mice is driven by the Acta2 promoter. Expression of the RAMP-1 mRNA was the same in both mouse lines with a non-significant tendency for reduction in response to hypertension. In contrast RAMP-2 expression was not only generally much less abundant but also significantly reduced by about 65% in both mouse lines in response to hypertension. Actb showed no expression changes in response to genetic alteration or chronic hypertension. Means ±SEM of 7 animals per group.

Expression analysis (cf. Table 1) of the CGRP and AM receptor components as well as the contractile proteins of the smooth muscle in the brain (Fig 5) revealed in normotensive mice, as expected, considerably higher Calclr expression in CLR-tg mice. Chronic hypertension induced in wt mice a significant suppression of Calclr mRNA (-70%) whereas it was slightly but non-significantly increased in hypertensive CLR-tg mice compared to their normotensive controls. This latter finding could be due to the hypertension-induced increase of the expression of Acta2 (significant in wt mice but not in CLR-tg mice) because the Acta2 promoter drives the transgenic overexpression of the Calclr in the CLR-tg mice. Generally, RAMP-1 expression was up to 10-times higher than RAMP-2 expression. During chronic hypertension RAMP-1 expression was slightly but non-significantly reduced (-28%) in both mouse lines. In contrast, compared to normotensive mice, RAMP-2 expression was clearly and significantly lower in hypertensive wt (-61%) as well as hypertensive CLR-tg mice (-66%). Regarding contractile protein expression in the brain extracts there was a considerable increase in smooth muscle alpha actin (Acta2) expression with chronic hypertension in line with others [26] of about 380% in wt and about 170% (non-significant) in CLR-tg mice. As another important contractile protein in smooth muscle we also determined β-actin (Actb) expression that was however not influenced by either genotype or hypertension.

## Discussion

Here we show that during chronic hypertension increased Calclr-signaling via CGRP receptors can maintain cerebrovascular reactivity despite an upward shift in the lower CA limit and the previously observed hypertension-induced increased smooth muscle alpha actin expression [26].

For humans it is known that women have lower systolic blood pressure levels than men especially during early adulthood [27,28]. In normal mice gender-dependent blood pressure differences appear to be quite strain dependent with higher systolic blood pressure observed sometimes in males but also in females [29]. In the present study we used mice with a C57BL6xDBA2 background and C57BL6 as well as DBA2 mice females have in tendency higher systolic blood pressure values than males [29].

Per definition essential (or primary) hypertension develops spontaneously without identified medical cause although there is a clear correlation with obesity and alcohol or tobacco consumption [30]. This form of hypertension accounts for 90–95% of all cases. Enormous progress has been achieved in the treatment of chronic essential hypertension but the clinical problems arising from the shift in cerebral blood flow auto-regulation towards higher blood pressure are not solved satisfactorily. For example, the advances of surgery and anesthesia allow operating on patients at much higher age and much lower general condition than in the past decades. However, this increases the risk and thus incidence of severe blood pressure disturbances during surgery and as a consequence brain damage because in chronic hypertensive patients CA, the important physiological mechanism that keeps CBF relatively constant in face of blood pressure changes, is shifted towards higher blood pressure values [5,7].

In our experiments 1K1C surgery induced an upward shift of the lower CA limit to the same extend in wt as well as CLR-tg mice. At the first glance this might suggest that enhancing the natural Calclr-expression of vascular smooth muscle cells [15] as in our CLR-tg mice [19,20] does not affect cerebrovascular reactivity during chronic hypertension. However, when determining the PDiVD (cf. Fig 1), there was a clear and significant effect of Calclr overexpression, namely that this genetic alteration is associated with a superior cerebrovascular reactivity (cf. Fig 3). Of note, this observation also suggests that merely determining the lower CA limit is not sufficient to judge cerebrovascular reactivity.

The molecular mechanisms of the RAMP expression and regulation are not entirely understood although the RAMP-1 gene expression might be mediated by a negatively acting transcription factor that represses RAMP-1 gene expression in RAMP-1 negative tissues [31]. Regarding the present study it is important to note that RAMP-2 expression is rapidly changed even upon small changes in systemic blood pressure. Decreases and increases of ±20–30 mmHg result in increases and decreases of RAMP-2 mRNA levels, respectively, in numerous brain structures whereas Claclr mRNA levels were not altered [32]. This suggests that the vasodilator responsiveness in hypertension at least to AM is regulated predominatly via the expression level of RAMPs and not the Calclr. Overexpression of the Calclr in smooth muscle cells should therefore capture newly produced RAMPs, form more surface receptors for CGRP and / or AM and, thus, increase the sensitivity of the vasculature to the respective peptide. To test this hypothesis we overexpressed in mice the Calclr in smooth muscle containing tissue, including the cerebral vasculature.

As the Calclr can associate with different RAMPs resulting in receptors for either CGRP or AM we wanted to assess which of these peptide is responsible for the better cerebrovascular reactivity in our CLR-tg mice. Unfortunately, the performance of commercially available antibodies for detection of CGRP and AM receptor components are in general raised against the human proteins and had poor performance on mouse brain sections. Therefore we used quantitative receptor autoradiography that in contrast to antibodies detects functional receptor complexes in order to compare the expression changes in CGRP and AM receptors in response to chronic hypertension. AM-receptor binding was not different between naive wt and CLR-tg but in both lines marginally although non-significantly reduced in response to chronic hypertension. In contrast, CLR-tg mice generally showed higher CGRP binding than wt mice and after induction of chronic hypertension we found a clear increase in CGRP-receptor binding in CLR-tg mice but only a trend in wt mice. Of note the striking down regulation of RAMP2 in parallel with unchanged RAMP1 expression suggests that CGRP receptors rather than AM receptors maintain cerebrovascular reactivity during chronic hypertension. Accordingly, autoregulatory vasodilatation in response to hypotension is attenuated by CGRP receptor desensitization and after application of capsaicin, which results in depletion of CGRP from perivascular neurons [10]. All these findings and also those of others suggest that, compared to AM, CGRP might play the more dominant role in the pathophysiology of chronic hypertension. In line with this interpretation it has been shown that CGRP plasma levels are lower in patients with essential hypertension and that plasma CGRP concentrations coincide with the nocturnal drop in systemic blood pressure [33]. However, this finding is in contrast to other studies reporting either unchanged [34] or even increased [35] CGRP plasma levels in hypertensive patients. These both latter findings could eventually be explained by impaired receptor function, which would fit to our observation of a reduced Calclr expression in hypertensive wt mice. In patients with phaeochromocytom the reduced blood pressure and CGRP plasma concentrations observed after adrenalectomy, however, indicate that CGRP secretion might be a compensatory mechanism to counteract hypertension [35]. Also in rats subjected to 2K1C surgery hypertension was associated with an elevated CGRP plasma level suggesting a compensatory role of this peptide in chronic hypertension [16]. Conversely, in phenol-induced hypertension the reduction of circulation CGRP contributes significantly to the elevated blood pressure [17]. Accordingly, in our experiments we found that CLR-tg mice developed slightly less severe hypertension 10 weeks after 1K1C surgery (cf. Fig 3B).

A previous study reported that overexpression of RAMP-1 augments cerebrovascular responses to CGRP and protects from angiotensin II-induced vascular dysfunction [36]. The authors conclude that CGRP-dependent cerebrovascular reactivity is RAMP-1-limited and, thus, not critically dependent on the Calclr expression. This is in accordance with our findings because the hypertension-induced drop of Calclr expression in wt mice was not associated with a comparable drop in AM or CGRP binding sites in the brain. The best explanation for this putative discrepancy is effective recycling of the Calclr. However, this is a difficult issue as the fate of the Calclr (recycling vs. degradation) after internalization depends on the receptor stimulation pattern (intermittent vs. sustained), fine-tuning of the expression of hepatocyte growth factor-regulated tyrosine kinase substrate (HRS) [37] and maybe other (even unknown) factors that could not be assessed in the present study. On the other hand, our data suggest that cerebrovascular reactivity might be also Calclr-limited as CLR-tg mice have during normotension as well as during chronic hypertension more CGRP binding sites in their brains. Of note, RAMP-1 expression was about 3.5-times higher compared to RAMP-2 expression in the normotensive state in both mouse lines and, more importantly, increased to a 6.6-times higher RAMP-1 expression over that of RAMP-2 due to a significant hypertension-induced drop in RAMP-2 expression. The latter is in accordance with a previous study on rats showing that RAMP-2 is down regulated upon short (within 6h) increases of blood pressure [32]. Here we show that this holds also for chronic hypertension and is most likely the primary cause for the increase in CGRP binding sites as long as enough Calclr is present as it is the case in CLR-tg mice but also in tendency in wt mice. This is an important notion because it demonstrates that naturally the hypertensive stimulus results in a strong reduction of RAMP-2 expression at an almost maintained RAMP-1 expression and therefore a shift of the cerebrovascular sensitivity ratio between CGRP and AM to CGRP. Overexpression of RAMP-1 [36] however univocally defines a higher abundance of the CGRP receptors on the cell surface and thus covers the natural hypertension-induced shift in the CGRP / AM sensitivity ratio of the cerebral vasculature that we describe here for the first time.

In summary our results suggest that chronic hypertension represses expression of all receptor components for CGRP as well as AM, however, the repression of RAMP-2 expression is strongest. As RAMP-2 expression is generally much lower than that of RAMP-1 the cerebrovascluar reactivity regarding CGRP and AM appears to rest mainly on CGRP and especially during chronic hypertension because this condition even more increases the expression ration of RAMP-1/RAMP2. Moreover chronic hypertension induces Acta2 [26] that is part of the contractile apparatus in smooth muscle cells. In this setting the vasoreactivity appears to be preserved as long as the CGRP receptors are upregulated. Thus, CGRP receptors play a significant role in maintenance of cerebrovascular reactivity during chronic hypertension, which should be kept in mind when treating hypertensive patients with CGRP antagonists as suggested e.g. for migraine [38].
